# Research on the impact of China’s reform to delegate power, streamline administration, and optimize government services on the technology innovation efficiency of the pharmaceutical manufacturing industry

**DOI:** 10.3389/fpubh.2024.1325298

**Published:** 2024-01-26

**Authors:** Yang Gu, Qian Zhuang

**Affiliations:** School of International Pharmaceutical Business, China Pharmaceutical University, Nanjing, China

**Keywords:** pharmaceutical industry, innovation efficiency, reform to delegate power, streamline administration and optimize government services, three-stage BCC model, inter-provincial

## Abstract

**Objective:**

The government has recently implemented reforms aimed at delegating power, streamlining administration, and optimizing government services. This reform has eliminated barriers that impede the growth of various industries, thereby unleashing innovative potential. Additionally, there have been several medical policies, including changes to medical insurance and centralized volume-based procurement. China’s pharmaceutical market has undergone significant changes, leading to increased demands for innovation technology efficiency in pharmaceutical manufacturing.

**Methods:**

The three-stage BCC theory was employed to assess the effectiveness of technology innovation in the industry under this reform. Calculate precise comprehensive technical efficiency values, pure technical efficiency values, and scale efficiency values for technological innovation in the pharmaceutical industry across 30 provinces from 2018 to 2020, after removing environmental factors.

**Results:**

In 2020, Jiangsu and Shandong and nine other provinces reached the comprehensive technical efficiency frontier surface, joining Tianjin, Zhejiang, and Guangdong provinces. However, Gansu, Qinghai, Ningxia, and Xinjiang still need to catch up due to their smaller industrial scale and lack of technology.

**Discussion:**

To ensure the effectiveness of reforms, it is crucial to fully consider provincial differences. Articulating national and provincial policies is necessary to allow efficient provinces to continue and allocate resources toward less efficient provinces to improve overall efficiency.

## 1 Introduction

China’s government has launched a new initiative called the deepening reform in delegating power, streamlining administration, and optimizing government services. The aim is to transform the functions of the government and reform the administrative system for the better. Since 2016, when the State Council held a national television and telephone conference to promote this initiative, the executive departments have been reducing direct intervention and focusing on macro-control, market supervision, and public services. This has helped to improve the business environment and stimulate innovation (1).

The pharmaceutical industry is crucial to people’s welfare and involves high-tech barriers. China’s pharmaceutical policies have been introduced intensively to address this industry’s special status. The Drug Administration, National Health Insurance Administration, and National Health and Health Commission are linked to reform and have been adjusting the base drug catalog, piloting DRG/DIP payment reform (2), promoting health insurance and health insurance fund reform, improving the traceability system and medical device management, carrying out the consistent evaluation of generic drug quality and efficacy, and organizing several national centralized drug volume-based procurements (3). The government encourages enterprises to research and develop innovations to improve quality and efficiency.

Premier Li Keqiang affirmed the effectiveness of the deepening reform in delegating power, streamlining administration, and optimizing government services at the first session of the 14th National People’s Congress, indicating that the changes in the industrial ecosystem, including the pharmaceutical manufacturing industry, have yielded positive benefits.

The pharmaceutical industry’s deepening reform can be achieved by encouraging pharmaceutical innovation. It is the most active and rapidly developing strategic new sector in the latest scientific and technological revolution. China needs to move from generic to innovative in the pharmaceutical field to achieve self-reliance in the high-tech industry.

The inefficiency of innovation caused by non-market factors in various regions of China, such as lengthy approval processes and blurred boundaries of authority and responsibility, needs to be addressed to deepen the reform in delegating power, streamlining administration, and optimizing government services. The government must clarify the relationship between the government and the market to release the industry’s creativity. The government and market should amend the unfavorable development of pharmaceutical industry innovation to ensure the safety and effectiveness of medical supplies and promote the high-quality development of the pharmaceutical manufacturing industry (4).

Internal problems of industrial innovation in China’s pharmaceutical industry also affect the improvement of innovation efficiency. There needs to be more basic theoretical research and high-quality innovations, more vigorous international discourse on medical standards, and research institutes focusing on practical application rather than just paper publication (5). The high cost, uncertainty, and failure rate in the new drug development process may also keep pharmaceutical companies with unfavorable financing away (6).

To promote the high-quality growth of the pharmaceutical manufacturing sector, this research empirically investigates the influence of the deepening reform in decentralizing power, simplifying administration, and enhancing government services. It identifies potential issues in each province that hinder the industry’s innovation efficiency and offers recommendations to resolve them.

## 2 Research methods

The operations research scientists Charnes, Cooper, and Rhodes (7) founded the data envelopment theory (DEA) in 1978. The first model of data envelopment theory, the radial CCR model, was named after the initials of their last names. The model connotes that, assuming constant returns to scale, the technical efficiency of each DMU(Decision-making unit), including scale efficiency, i.e., the comprehensive technical efficiency, is calculated according to input orientation or output orientation. Another feature of the radial DEA model is that the inefficient decision unit is improved by scaling down (increasing) all inputs (outputs) equally. With the CCR model’s application in management and economics, the initial CCR model revealed some defects and shortcomings. Banker et al. (8) proposed a radial BCC model based on variable payoffs of scale, which can exclude the influence of scale efficiency technical efficiency, known as pure technical efficiency, which is more in line with the actual situation where many production units are not in the production state of optimal scale. The radial DEA model is one of the types of distance function DEA in improving inefficient decision units. Other types of distance function DEA have been proposed by scholars in recent years, such as the distance to frontier furthest position (SBM model) proposed by Kaoru et al. (9), directional distance function (DDF model) proposed by Chung et al. (10), etc.

Stochastic Frontier analysis (SFA) is another way to measure the technical efficiency of DMU. Based on the deterministic production frontier, the SFA model proposed by Aigner et al. (11). Estimates the technical efficiency of DMU by decomposing the error term into random error and technical inefficiency. Fried (12) effectively eliminated input or output variables containing environmental factors and random noise by selecting environmental indicators and reintroduced the eliminated input or output into the DEA model to obtain more accurate technical efficiency. According to Fried’s research method, considering that inter-provincial governments will have different effects in deepening reform in delegating power, streamlining administration, and optimizing government services, there will be differences in the development of the pharmaceutical manufacturing industry.

Cornejo (13) uses a dynamic output-oriented DEA (DNSBM) with a network structure based on the SBM framework to assess whether European countries are effectively managing their R&D and innovation resources. Guo (14) studied the efficiency of green innovation in Northeast China from 2005 to 2020 using the DEA model (EBM-Malmquist). The study found evidence of a cyclical cumulative effect of green innovation. Liu’s conclusions find that innovation input carbon intensity is the primary factor inhibiting the growth of carbon emissions (15). Zhao (16) came to some interesting conclusions; the level of technological innovation drove the green transition in manufacturing, but research investment did not have the expected positive effect, the level of economic development had a negative effect on the green transition, and industrialization and urbanization had a positive effect on efficiency.

Fu (17) developed a four-stage NDDF-DEA model for a study of 30 selected provinces in China and concluded that digital transformation improves agro-ecological efficiency by promoting technological innovation. Zhang and Fu (18) to analyze the role of technological progress in improving energy efficiency. The first stage consists of estimating the Total Factor Energy Efficiency (TFEE) scores using the Data Envelopment Analysis (DEA) method of super-efficiency, while the second stage consists of exploring how technological innovation and Foreign Direct Investment (FDI) affect energy efficiency.

The above measures of efficiency shed light on the stakeholders, in particular the government or trade associations. In this study, the input-oriented three-stage BCC model was used to evaluate the pharmaceutical manufacturing industry’s scientific and technological innovation efficiency in 30 provinces under the background of deep reform.

### 2.1 First stage

The input-oriented BCC model was used to analyze the original input–output of each province from 2018 to 2020. The comprehensive technical efficiency (TE) is obtained. Decomposition of the integrated technical efficiency yields scale efficiency (SE) and pure technical efficiency (PE). Due to the variable scale condition, the product of PE and SE is equal to TE. The planning equation of the input-oriented BCC model is given in Equation 1 (19).


(1) $$\begin{array}{c} \max Y_{k}=\overset{q}{\underset{r=1}{\sum }}\;\lambda_{r}y_{rk}-\mu_{k}\\ \begin{array}{c} \;s.t.\overset{q}{\underset{r=1}{\sum }}\;\theta_{i}x_{ik}=1\\ \overset{q}{\underset{r=1}{\sum }}\;\lambda_{r}y_{rk}-\overset{q}{\underset{r=1}{\sum }}\;\theta_{t}x_{ik}-\mu_{k}\leq 0\\ \lambda_{r}\geq 0,\theta \geq 0\\ k=1,2,\cdots ,n;r=1,2,\cdots ,q;i=1,2,\cdots ,m \end{array}\; \end{array}$$

In Equation 1, k indicates that the number of DMU is n; i and r indicate that each DMU has m inputs and q outputs, respectively; the technical efficiency of the k-th DMU is denoted as Y_k_; x_ik_ and y_rk_ are the i-th input and r-th output of the k-th DMU, respectively; θ_i_ is the weight of the i-th input; λ_r_ is the weight of the r-th output. The BCC model calculates the amount of slack in the input–output variables of each province, the preliminary comprehensive technical efficiency, pure technical efficiency, and scale efficiency.

### 2.2 Second stage

According to the method proposed by Fried, the four input slack variables calculated in the first stage are used as explanatory variables in this research. The selected environmental variables are the explanatory variables. Thus, four stochastic frontier analysis (SFA) models were constructed. The SFA models are shown in Equation 2.


(2) $$\begin{array}{c} S_{ki}=f\left(Z_{k};\beta_{i}\right)+v_{ki}+u_{ki}\;\\ i=1,2,\cdots ,m;k=1,2,\cdots ,n\; \end{array}$$

In Equation 2, S_ki_ is the slack value of the i-th input of the k-th DMU; Z_k_ is the environmental variable; β_i_ is the coefficient of the environmental variable; v_ki_ is the random disturbance, u_ki_ is the management inefficiency, and the sum of random disorder and management inefficiency is the random error term (v_ki_ + u_ki_). Since the premise of using the stochastic frontier model is the existence of management inefficiency u, this study uses a one-sided generalized likelihood ratio test to make it obey in a half-normal distribution on the basis of the test existence.

According to the regression results of the SFA model, the DMU is placed in the same external environment with random disturbances to adjust the original input quantity. The input variable adjustment is shown in Equation 3.


(3) $$X_{ki}^{A}=X_{ki}+\left[\max\left(f\left(Z_{i};{\hat{\beta }}_{k}\right)\right)-f\left(Z_{i};{\hat{\beta }}_{k}\right)\right]+\left[\max\left(v_{ki}\right)-v_{ki}\right]$$

In Equation 3, with reference to Jondrow, constructing $\left[\max\left(f\left(Z_{i};{\hat{\beta }}_{k}\right)\right)-f\left(Z_{i};{\hat{\beta }}_{k}\right)\right]$ , which indicates adjusting the DMU to the same environmental factors; constructing $\left[\max\left(v_{ki}\right)-v_{ki}\right]$ , which indicates adjusting the DMU to the same stochastic conditions; and X, which indicates the adjusted inputs (20).

### 2.3 Third stage

The adjusted input variables replaced the initial input variables in the second stage. The BCC model is used to calculate and decompose the adjusted innovation inputs and outputs again. The comprehensive technical efficiency, pure technical efficiency, and scale efficiency, which exclude the three effects of management inefficiency, random interference, and external environment, are finally obtained.

## 3 Construction of evaluation index system

### 3.1 Input indicators

As shown in Table 1, in terms of the government’s practical actions to reduce micro-management and direct intervention and focus on strengthening macro-regulation, market supervision, and public services, this research selects two indicators that can indicate the effect of the deepened reform in delegating power, streamlining administration, and optimizing government services, which are the indicator of government funding subsidies in enterprises’ internal R&D expenditures (referred to as Subsidy). The hand of inter-provincial Medical Products Administration accounts expenses (referred to as Account).

**Table 1:** Index system.

Index system	Index name	Index meaning	Data source	Calculation method	Unit
Input	Subsidy	Government funding subsidies	EPSDATA Database	Direct statistics	10 K CNY
	Account	Regulatory account expenditures	Official Website of Pro-Governments	Direct statistics	10 K CNY
	Material	The cost of R&D of new products in the firm	EPSDATA Database	Direct statistics	10 K CNY
	Intelligence	The number of R&D personnel in the firm	EPSDATA Database	Direct statistics	Per person
Output	Patent	No. of patent applications for inventions	EPSDATA Database	Direct statistics	Pieces
	Sales	Revenue from sales of new products	EPSDATA Database	Direct statistics	10 K CNY
	project	Number of new projects established	EPSDATA Database	Direct statistics	Pieces
Environmental	People’s welfare	*Per capita* disposable income	EPSDATA Database	Direct statistics	0.01 K CNY
	Industrial development	Fixed Assets of Industrial Enterprises Above Scale	EPSDATA Database	Direct statistics	10 K CNY
	Economic level	Gross Domestic Product (GDP)	EPSDATA Database	Direct statistics	0.01 K CNY
	Talent education	Number of PhD graduates	EPSDATA Database	Direct statistics	Per person

The reason for choosing the indicator of government funding subsidies in enterprises’ internal R&D expenditures is that, as mentioned earlier, scientific and technological innovation in pharmaceutical enterprises is extremely risky and high-cost, which requires the government to strengthen macro-regulation and service work. The pharmaceutical industry in science and technology innovation boosts confidence. Breaking down the linkage between government departments and the pharmaceutical manufacturing industry, the provincial Medical Products Administration is responsible for the vast majority of the management and supervision of pharmaceutical enterprises in the province, from R&D to production. Its final account funds are closely related to enterprise science and technology innovation. There is reasonableness in choosing this indicator.

Based on enterprise science and technology innovation conditions, this Study selects indicators in two aspects of intellectual input and material input in the pharmaceutical manufacturing industry. In terms of academic input, the R&D personnel of pharmaceutical manufacturing enterprises are selected. This indicator can fully reflect the total Intelligence engaged in 3 activities: basic research, applied research, and experimental development within the enterprise(referred to as Intelligence). Regarding material investment, the expenditure on new product development of pharmaceutical manufacturing enterprises is selected. This indicator is the sum of spending on research, design, model development, testing, and experiment of new products, which can well reflect the intensity of enterprises’ scientific and technological innovation activities (referred to as Material).

### 3.2 Output indicators

As shown in Table 1, based on the possible outputs of science and technology innovation activities. We are drawing on the experience of selecting innovation efficiency indicators by scholars such as Chen Yuwen. This research includes the number of patent applications, new product sales revenue, and new product development projects of pharmaceutical manufacturing enterprises as the output indicators of science and technology innovation (21).

The indexes of the number of patents applied for inventions in pharmaceutical manufacturing enterprises reflect the innovation dynamics and degree of pharmaceutical enterprises (referred to as Patents). The new product sales revenue indicator indicates the efficiency of innovation transformation and innovation profit of pharmaceutical enterprises (referred to as Sales). The number of new product development projects indicates the future development trend and innovative means of pharmaceutical enterprises to grasp the increasingly fierce market competition (referred to as Project).

### 3.3 Environmental indicators

We select the environmental indicators in this research according to their substantial impact on the innovation efficiency of pharmaceutical enterprises and the government’s deepening reform. However, it can not be controlled by a single administrative means or enterprise behavior. The pharmaceutical manufacturing industry is one of the six high-tech industries in China, and its innovation input and output activities are closely related to four aspects: talent education, economic level, people’s welfare, and industrial development, in addition to the government’s deepening reform in delegating power, streamlining administration and optimizing government services (Table 1).

#### 3.3.1 Talent education

In developing high-quality innovation in the pharmaceutical manufacturing industry, scientific and technological progress plays the “locomotive.” And scientific and technological progress depends on talent. Talent training relies on education. The talent education system of the whole society is the cornerstone for the whole pharmaceutical manufacturing industry to obtain cutting-edge ideas & science & technology. It is also the result of the government’s continuous adjustment and optimization of the educational structure. Therefore, it is reasonable to include talent education in the environmental indicators. This research selects the number of inter-provincial higher education doctoral graduates (graduates) to characterize talent education in each province.

#### 3.3.2 Economic level

The inter-provincial economic level is affected by several factors, one of which is the effectiveness of the government’s deepening reform in delegating power, streamlining administration, and optimizing government services. Given the scientific nature of Xia Huiliang et al.’s study, this study also selects the inter-provincial GDP *per capita* to characterize the economic level of each province.

#### 3.3.3 People’s welfare

The medical reform in recent years has been quite dynamic. The pharmaceutical policy, represented by the adjustment of the essential drug catalog and China’s centralized Volume-Based Procurement, has effectively reduced the burden of medication on the public and gradually led to the return of drug prices to a reasonable level. The pharmaceutical industry is unique, and the prices of drugs and other products must be linked to the public’s affordability and ability to pay. Hence, this paper selects inter-provincial cities’ *per capita* disposable income to characterize the people’s welfare.

#### 3.3.4 Industrial development

Pharmaceutical manufacturing is one of the industrial categories. It is closely related to other industries, such as logistics, chemical instrument manufacturing, etc. The Investment in fixed assets of industry promotes the construction of the pharmaceutical manufacturing industry chain, guarantees manufacturing and supply, and helps innovation in the pharmaceutical industry. In this research, the total fixed assets of industrial enterprises above the provincial scale are selected to indicate industrial development in each province.

### 3.4 Correlation test of indicators

A Pearson correlation test was conducted on the selected indicators to ensure the scientific validity of the input and output indicators. This test aimed to determine if there is a positive correlation between the indicators (22). The results show that all input indicators are significantly correlated with output indicators at the 1% level (two-sided) and are positively correlated, which satisfies the correlation requirement of the DEA model for indicators. We show the test results in Table 2.

**Table 2:** Pearson’s correlation test.

Outputs\Inputs	Subsidy	Material	Account	Intelligence
Patent	0.824***	0.927***	0.508***	0.939***
Sales	0.759***	0.957***	0.460***	0.947***
project	0.820***	0.895***	0.423***	0.956***

Obtained by SPSS26 software processing. ***Indicates significant at the 1% level.

## 4 Empirical analysis

### 4.1 First stage BCC empirical analysis

Based on whether the government’s deepening reform in delegating power, streamlining administration, and optimizing government services impacts the efficiency of science and technology innovation in the pharmaceutical manufacturing industry. In the first stage of the study, the environmental factors and random error terms were not considered. The initial input–output variables of each province were calculated with the help of Dearun 3.0 software. The comprehensive technical efficiency (TE), scale efficiency (SE), and pure technical efficiency (PE) of the pharmaceutical manufacturing industry were obtained, where TE = SE * PE. From the perspective of comprehensive technical efficiency, the government’s deepened reform in delegating power, streamlining administration, and optimizing government services has significantly improved the efficiency of scientific and technological innovation in the pharmaceutical manufacturing industry. The efficiency of the national, eastern, western, central, and northeastern regions has been improved. By 2020, only the comprehensive technical efficiency of the western region is below 0.9, as shown in Table 3.

**Table 3:** Comprehensive technical efficiency of the first stage.

Region	Comprehensive technical efficiency			Trends for 2018–2019	Trends for 2019–2020
	2018	2019	2020		
National	0.79	0.85	0.91	↗	↗
Eastern	0.78	0.86	0.93	↗	↗
Beijing	0.70	0.84	0.94	↗	↗
Tianjin	0.98	1.00	1.00	↗	↗
Hebei	0.71	1.00	1.00	↗	↗
Shanghai	0.72	0.81	0.63	↗	↘
Jiangsu	0.76	0.69	1.00	↘	↗
Zhejiang	0.98	0.91	0.87	↘	↘
Fujian	0.69	0.68	0.84	↘	↗
Shandong	0.76	0.87	0.97	↗	↗
Guangdong	0.79	0.89	1.00	↗	↗
Hainan	0.75	0.93	1.00	↗	↗
Central	0.79	0.87	0.95	↗	↗
Shanxi	0.97	0.92	1.00	↘	↗
Anhui	0.98	1.00	1.00	↗	↗
Jiangxi	0.72	0.87	1.00	↗	↗
Henan	0.58	0.62	0.67	↗	↗
Hubei	0.67	0.86	1.00	↗	↗
Hunan	0.81	0.92	1.00	↗	↗
Western	0.78	0.80	0.87	↗	↗
Neimenggu	0.57	0.89	0.74	↗	↘
Guangxi	1.00	1.00	1.00	↗	↗
Chongqing	0.97	0.97	0.98	↗	↗
Sichuan	0.77	0.81	0.98	↗	↗
Guizhou	0.94	1.00	1.00	↗	↗
Yunnan	0.80	0.83	0.86	↗	↗
Shaanxi	0.55	1.00	1.00	↗	↗
Gansu	0.45	0.37	0.52	↘	↗
Qinghai	1.00	0.56	1.00	↘	↗
Ningxia	0.78	0.56	0.58	↘	↗
Xinjiang	0.75	0.85	0.92	↗	↗
Northeast	0.87	0.95	0.98	↗	↗
Liaoning	1.00	1.00	1.00	↗	↗
Jilin	0.62	0.87	0.94	↗	↗
Heilongjiang	0.99	0.99	1.00	↗	↗

**Figure 1:**
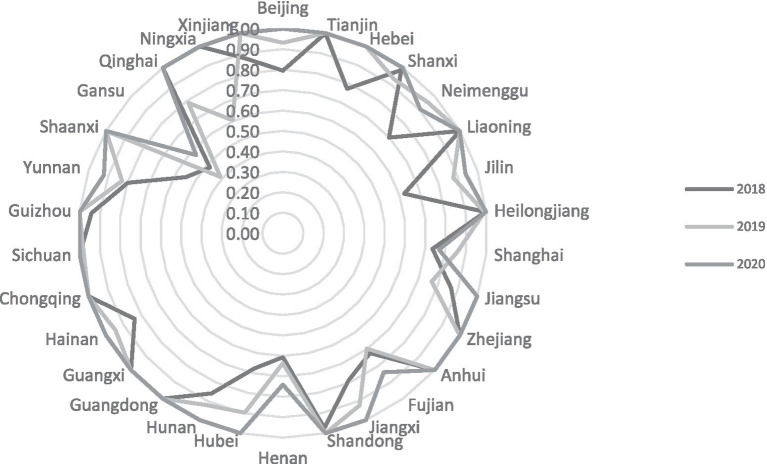
Pure technical efficiency of the first stage.

From a purely technical efficiency perspective, numerous provinces were below 0.8 in 2018. It got an overall improvement in the following two years. In addition, the pure technical efficiency of the traditionally cognitively strong pharmaceutical provinces represented by Zhejiang, Jiangsu, and Shandong in all years is significantly better than that of most provinces, such as Ningxia and Gansu, as shown in Figure 1.

**Figure 2:**
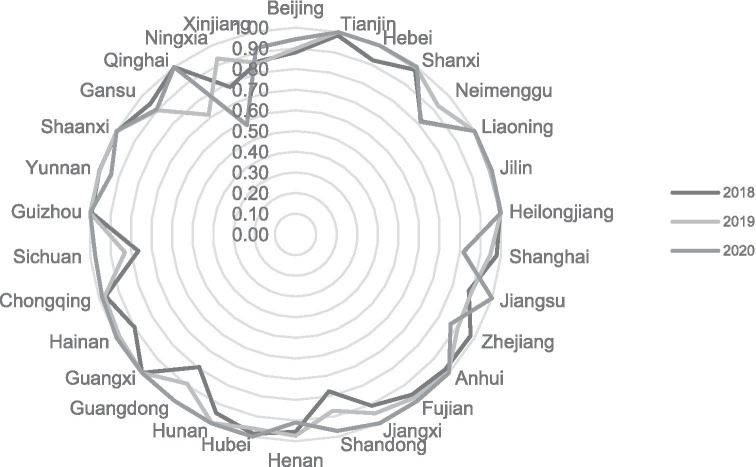
Scale efficiency of the first stage.

Overall, scale efficiency, excluding Ningxia and Qinghai provinces, annual fluctuations are relatively small and stable. The government’s deepening reform in delegating power, streamlining administration, and optimizing government services has increased the scale efficiency by a small margin year by year, and compared the comprehensive technical efficiency and pure technical efficiency, found that the scale efficiency is generally high, most in the effective frontier surface. We show this outwork in Figure 2.

### 4.2 Second stage SFA empirical analysis

If there are environmental factors and random errors, there will be some deviation in each efficiency value calculated by the first stage BCC model. The regression results of the four SFA equations constructed by Frontier 4.1 software are shown in Table 4. The LR one-sided test for all four models reached a 5% or 1% significance test.

**Table 4:** SFA regression analysis of the second stage.

Environmental variables	Input slack-Subsidy	Input slack-material	Input slack-account	Input slack-intelligence
Constant term	2089.32**	−580989.09***	−346948.46***	7978.66***
People’s welfare	−1262.90***	165750.37***	98333.51***	−3024.27***
Industrial development	−65.21	−13474.32***	−13215.00***	−83.01
Economic level	805.52**	−30432.12***	−15501.76***	1199.57***
Talent education	−5.93	6474.55***	5609.91***	86.09
σ^2	131515.43***	491039440.00***	214534320.00***	443779.65***
γ	0.43***	0.54***	0.73***	0.47***
LR	9.44**	12.18**	42.15***	23.27***

** and *** represent significant at the level of 5% and 1%, respectively.

Environmental variables such as people’s welfare, industrial development, economic level, and talent education significantly affect inter-provincial deep reform’s input slackness in delegating power, streamlining administration, and optimizing government services. It also significantly affects the amount of input slack in inter-provincial pharmaceutical manufacturing technology innovation.

The values of input slack in the four models passed the 1% significance test (γ), indicating the existence of management inefficiency and random interference. The SFA model was constructed to remove the effects of management inefficiency and random interference on the efficiency of science and technology innovation in the pharmaceutical manufacturing industry under the government’s deepening reform in delegating power, streamlining administration, and optimizing government services.

It is worth noting that there are two kinds of correlation between the slack of input variables and environmental factors. When the coefficient of the environmental factor is positive, it indicates a positive promoting effect on the input slack. With the development of this environmental factor, enhanced input will lead to input waste or output decline. Similarly, when the coefficient of the environmental factor is negative, the environmental factor will negatively inhibit the input slack. The development of environmental factors reducing the input will promote the increase of output. The specific influences of environmental factors selected in this research on the slack amount of each input are:

*People’s welfare*: This environmental factor negatively correlates with Input slack-subsidy and Input slack-intelligence at above 1%. It indicates that improving People’s welfare will improve the efficiency of government funding investment and enterprise R&D personnel input and output. The reason may be that high People’s welfare will have a solid attraction to R&D personnel at all levels, and the government will pay more attention to the development of related industries and invest more funds to subsidize them, creating a synergistic development effect. In addition, People’s welfare is positively correlated with the Input slack material, and the Input slack account at the 1% level, indicating that the increase in People’s welfare is not conducive to the input–output efficiency of enterprise innovation development expenditure and Medical Products Administration in various places. From the perspective of incentive theory, high People’s welfare generates a certain degree of incentive dysfunction and management failure.

*Industrial development*: This environmental factor is negatively correlated with Input slack material, and the Input slack accounts at the 1% level, indicating that industrial development helps the government to improve the efficiency of settlement and enterprise innovation development expenditure, generating a synergistic and positive development. An excellent industrial environment benefits the micro subjects of technological innovation in the pharmaceutical industry, such as the government and enterprises.

*Economic level*: This environmental factor is negatively correlated with the Input slack-material and the Input slack account at the 1% level, indicating that the higher the economic level of each place, the less wasteful the enterprise development expenditure and the final account of the Medical Products Administration, and the more beneficial to industrial innovation. In addition, the economic level is positively associated with the Input slack subsidy and the Input slack intelligence at the 5 and 1% levels, respectively, which is consistent with the findings of Cao Yang et al. Higher economic levels could lead to waste of government funding subsidies and R&D personnel input resources, reducing scientific and technological innovation efficiency in the pharmaceutical manufacturing industry.

*Talent education*: This environmental factor is positively correlated with the Input slack material and the Input slack account at the 1% level, indicating that although the improvement of talent education can increase innovation and workforce in the pharmaceutical manufacturing industry, the poor allocation of talent education to corporate development and pharmacovigilance sector funding can produce substantial inefficiencies. The efficiency of the transformation of talent advantage is a powerful driving force for China’s high-quality development in the future.

### 4.3 Third stage efficiency value analysis

#### 4.3.1 Inter-provincial innovation efficiency development trend analysis

The SFA regression results in the second stage show that environmental factors and random errors influence the original input–output data. The adjusted inputs and outputs were calculated again by the BCC model based on a variable scale. We obtained more accurate comprehensive technical efficiency (TE), scale efficiency (SE), and pure technical efficiency (PE) of pharmaceutical manufacturing industry innovation under the deepening reform in delegating power, streamlining administration, and optimizing government services, TE = SE * PE.

The efficiency results of the third stage are shown in Table 5. The comprehensive technical efficiency, scale efficiency, and pure technical efficiency of the country’s four regions, east, west, central, and northeast, increased year by year. The deepening reform in delegating power, streamlining administration, and optimizing government services has indeed driven the improvement of science and technology innovation in the pharmaceutical manufacturing industry. In terms of pure technical efficiency, all regions are at a high level. However, in terms of scale efficiency, there are significant differences; the scale efficiency of the eastern region in three years was 0.93, 0.92, 0.96; the central region is the next, 0.86, 0.85, 0.94; the northeast region again: 0.73, 0.77, 0.78; the lowest scale efficiency is the western region, only 0.50, 0.53, 0.58. Scale efficiency of the four regions Efficiency comparison, reflecting the imbalance of China’s pharmaceutical manufacturing industry agglomeration, the scale gap between the regional pharmaceutical industry is obvious, hindering the development of pharmaceutical manufacturing innovation in the western region and other regions.

**Table 5:** Stage 3 efficiency.

Region	TE			SE			PE		
	2018	2019	2020	2018	2019	2020	2018	2019	2020
National	0.68	0.71	0.79	0.74	0.75	0.80	0.92	0.96	0.98
Eastern	0.86	0.88	0.95	0.93	0.92	0.96	0.92	0.96	0.98
Beijing	0.75	0.82	1.00	0.87	0.87	0.92	0.86	0.95	1.00
Tianjin	1.00	1.00	1.00	1.00	1.00	1.00	1.00	1.00	1.00
Hebei	0.79	0.93	1.00	0.94	0.94	1.00	0.84	1.00	1.00
Shanghai	0.72	0.75	0.78	0.88	0.83	0.94	0.83	0.91	0.84
Jiangsu	0.87	0.79	1.00	1.00	0.98	1.00	0.88	0.81	1.00
Zhejiang	1.00	1.00	1.00	1.00	1.00	1.00	1.00	1.00	1.00
Fujian	0.76	0.79	0.81	0.85	0.87	0.83	0.89	0.91	0.97
Shandong	0.97	1.00	1.00	0.99	1.00	1.00	0.97	1.00	1.00
Guangdong	1.00	1.00	1.00	1.00	1.00	1.00	1.00	1.00	1.00
Hainan	0.69	0.70	0.91	0.74	0.72	0.91	0.94	0.98	1.00
Central	0.75	0.79	0.91	0.86	0.85	0.94	0.88	0.93	0.97
Shanxi	0.59	0.63	0.82	0.60	0.65	0.82	0.98	0.97	1.00
Anhui	0.94	0.89	0.99	0.94	0.89	0.99	1.00	1.00	1.00
Jiangxi	0.82	0.87	1.00	0.95	0.93	1.00	0.86	0.94	1.00
Henan	0.62	0.65	0.76	0.86	0.87	0.95	0.72	0.75	0.80
Hubei	0.72	0.89	1.00	0.90	0.93	1.00	0.79	0.95	1.00
Hunan	0.82	0.79	0.90	0.90	0.82	0.90	0.91	0.96	1.00
Western	0.47	0.51	0.58	0.50	0.53	0.58	0.95	0.97	0.98
Neimenggu	0.27	0.35	0.43	0.29	0.36	0.44	0.92	0.98	0.96
Guangxi	0.49	0.49	0.58	0.49	0.49	0.58	1.00	1.00	1.00
Chongqing	0.91	0.89	0.90	0.91	0.89	0.90	1.00	1.00	1.00
Sichuan	1.00	0.96	1.00	1.00	0.98	1.00	1.00	0.98	1.00
Guizhou	0.64	0.69	0.70	0.66	0.70	0.70	0.96	0.99	1.00
Yunnan	0.69	0.67	0.79	0.76	0.72	0.81	0.91	0.94	0.97
Shaanxi	0.48	0.77	0.95	0.59	0.77	0.95	0.81	1.00	1.00
Gansu	0.24	0.25	0.30	0.28	0.30	0.34	0.85	0.84	0.90
Qinghai	0.16	0.13	0.16	0.16	0.14	0.16	1.00	0.98	1.00
Ningxia	0.17	0.18	0.23	0.18	0.19	0.24	0.98	0.95	0.98
Xinjiang	0.17	0.26	0.30	0.17	0.26	0.30	0.98	1.00	1.00
Northeast	0.69	0.76	0.77	0.73	0.77	0.78	0.94	0.98	0.98
Liaoning	0.59	0.60	0.63	0.59	0.60	0.63	1.00	1.00	1.00
Jilin	0.54	0.68	0.68	0.67	0.72	0.72	0.81	0.94	0.95
Heilongjiang	0.93	1.00	1.00	0.94	1.00	1.00	1.00	1.00	1.00

Compared with the scale efficiency in the first stage, the efficiency of most provinces is significantly reduced. The reason for this is to remove the influence of environmental factors and random disturbances so that the efficiency values of each province are close to the actual development of the province. For example, the three-year scale efficiency of Xinjiang before adjustment is 0.86, 0.85, and 0.92 in order, and the three-year scale efficiency of Xinjiang after adjustment is 0.17, 0.26, and 0.30 in order. Although both can show the trend of efficiency progress, the substantive meaning differs. It shows that the scale is one of the key factors impeding the further improvement of the efficiency of science and technology innovation in the pharmaceutical manufacturing industry in Xinjiang.

Similar scale factors constrain the efficiency of innovation in the pharmaceutical manufacturing industry under the deepening reform in delegating power, streamlining administration, and optimizing government services in provinces such as Guizhou, Shaanxi, Gansu, Qinghai, Ningxia, Inner Mongolia, Liaoning, and Jilin. There are still some provinces with industry technology level that is not high, low technology content, or poor resource allocation. This has led to the province’s deep reform in delegating power, streamlining administration, and optimizing government services of the pharmaceutical manufacturing industry. Innovation efficiency could be more precise in Henan, Shaanxi, Hebei, and other provinces. Compared with the three years of efficiency development, Hubei Province and Jiangxi Province showed a promising trend of technological progress and scale growth, with comprehensive technical efficiency from 0.72 to 0.82 intermediate level. Finally, they reached 1 in 2020, at the forefront of technology. In the need for more development of various efficiencies in the whole western region, Sichuan and Chongqing have a large-scale pharmaceutical manufacturing industry and a high level of technology, which plays a central role in the region.

Jiangsu, Zhejiang, Beijing, Tianjin, Shandong, Guangdong, and 11 other provinces (municipalities directly under the Central Government) in 2020 to reach the technological frontier surface, the best allocation of existing resources, the deepen reform in delegating power, streamlining administration and optimizing government services is effective, the enterprise rejuvenated innovation, improve the level of technology and scale of the pharmaceutical manufacturing industry, there is advanced experience, worth Other provinces to learn.

Table 5 shows that the reform to delegate power, streamline administration, and optimize government services has yielded significant benefits for China. The increase in comprehensive technical efficiency values from 0.68 to 0.79, scale efficiency values from 0.74 to 0.80, and pure technical efficiency values from 0.92 to 0.98 provides evidence of this.

**Figure 3:**
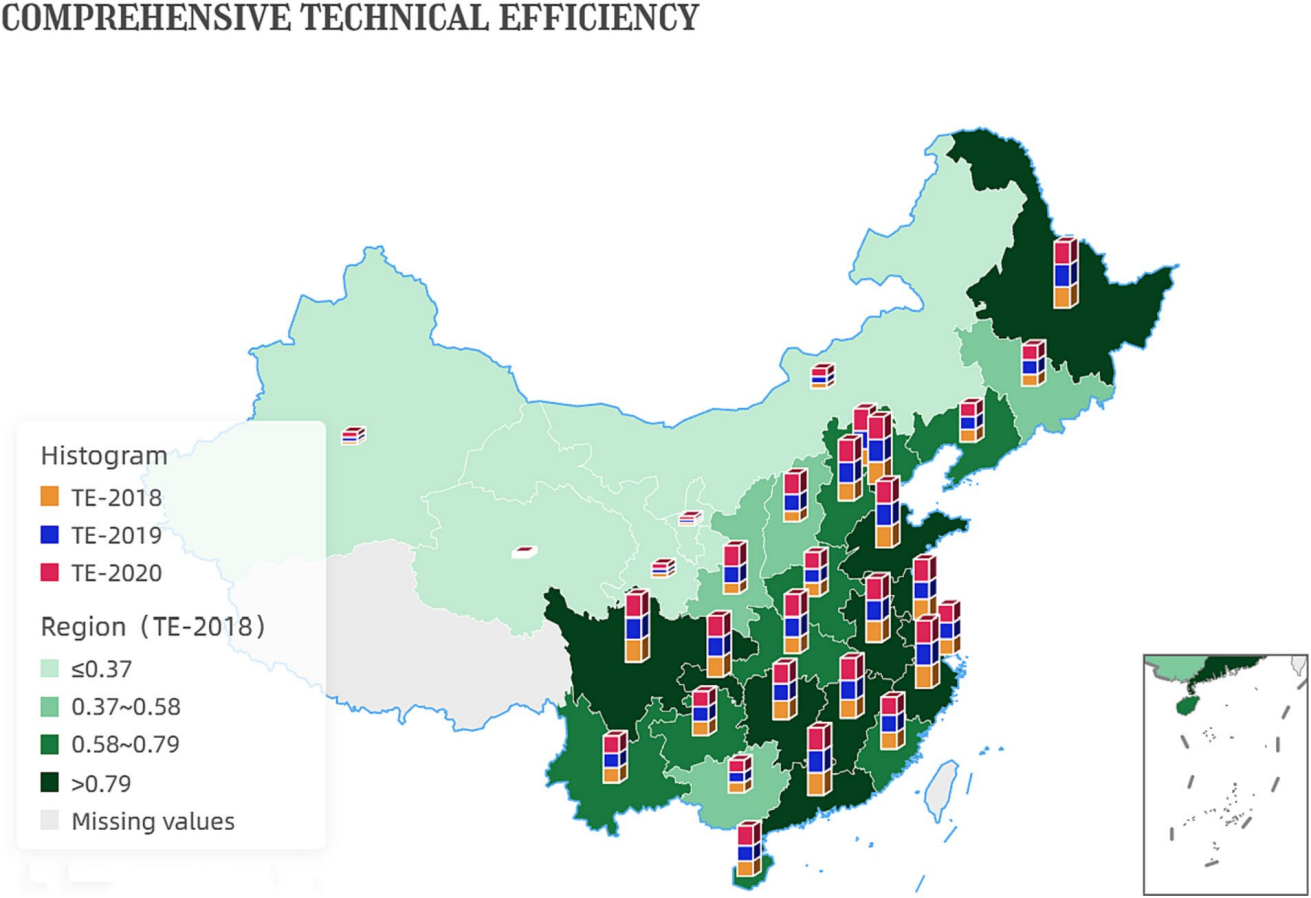
Development of inter-provincial comprehensive technical efficiency.

These numbers indicate a positive trend in the efficiency of China’s government services and administrative processes. The increase in comprehensive technical efficiency values suggests that the productivity and effectiveness of the government have improved. This could be due to better allocation of resources, improved management practices, or more effective policies and regulations. Figure 3 shows the development of inter-provincial comprehensive technical efficiency.

**Figure 4:**
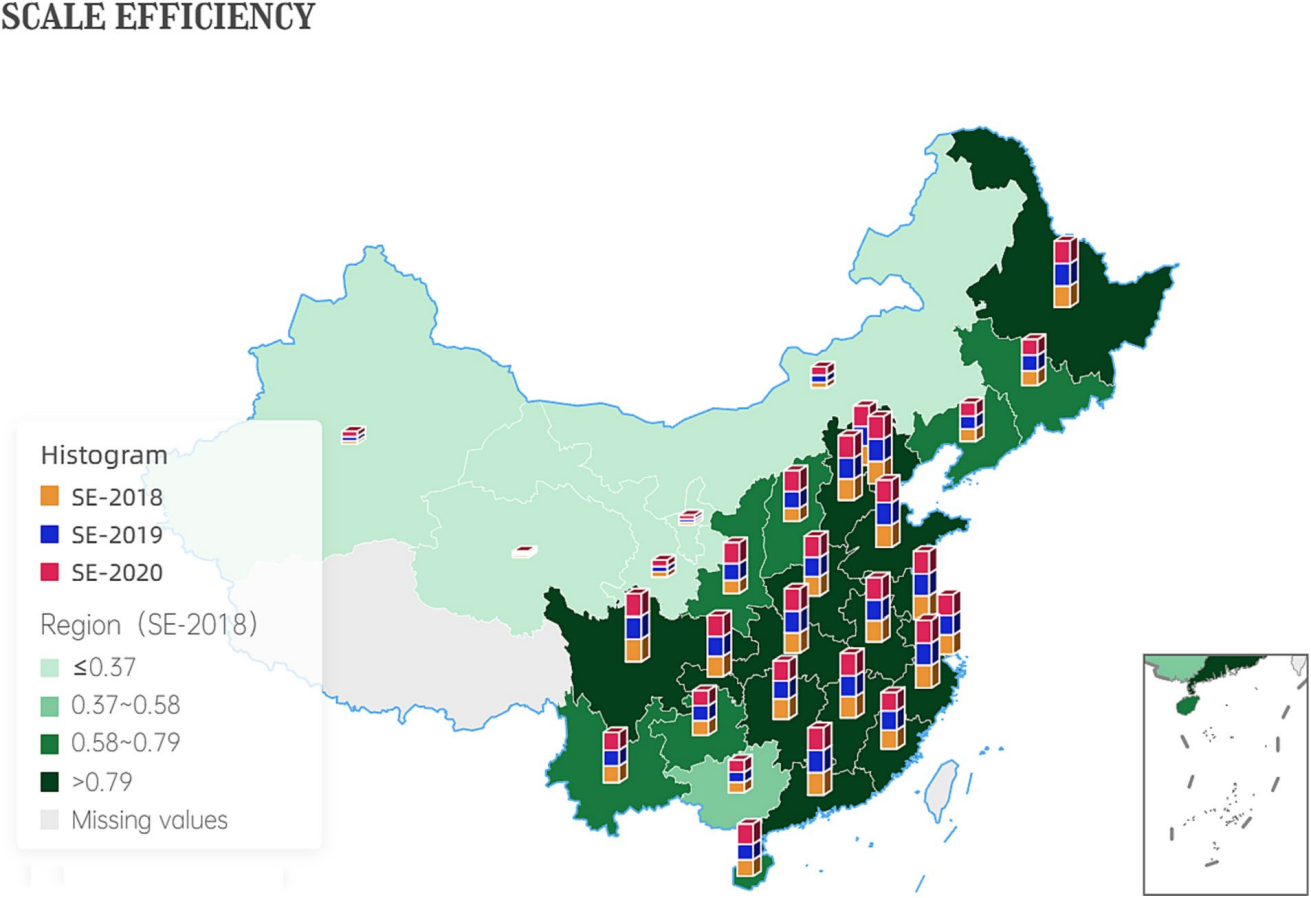
Development of inter-provincial scale efficiency.

The increase in scale efficiency values suggests that the government has achieved higher levels of output per unit of input, indicating increased productivity. This could be due to economies of scale, improved resource allocation, or more efficient use of technology and infrastructure. Figure 4 shows the development of inter-provincial scale efficiency.

**Figure 5:**
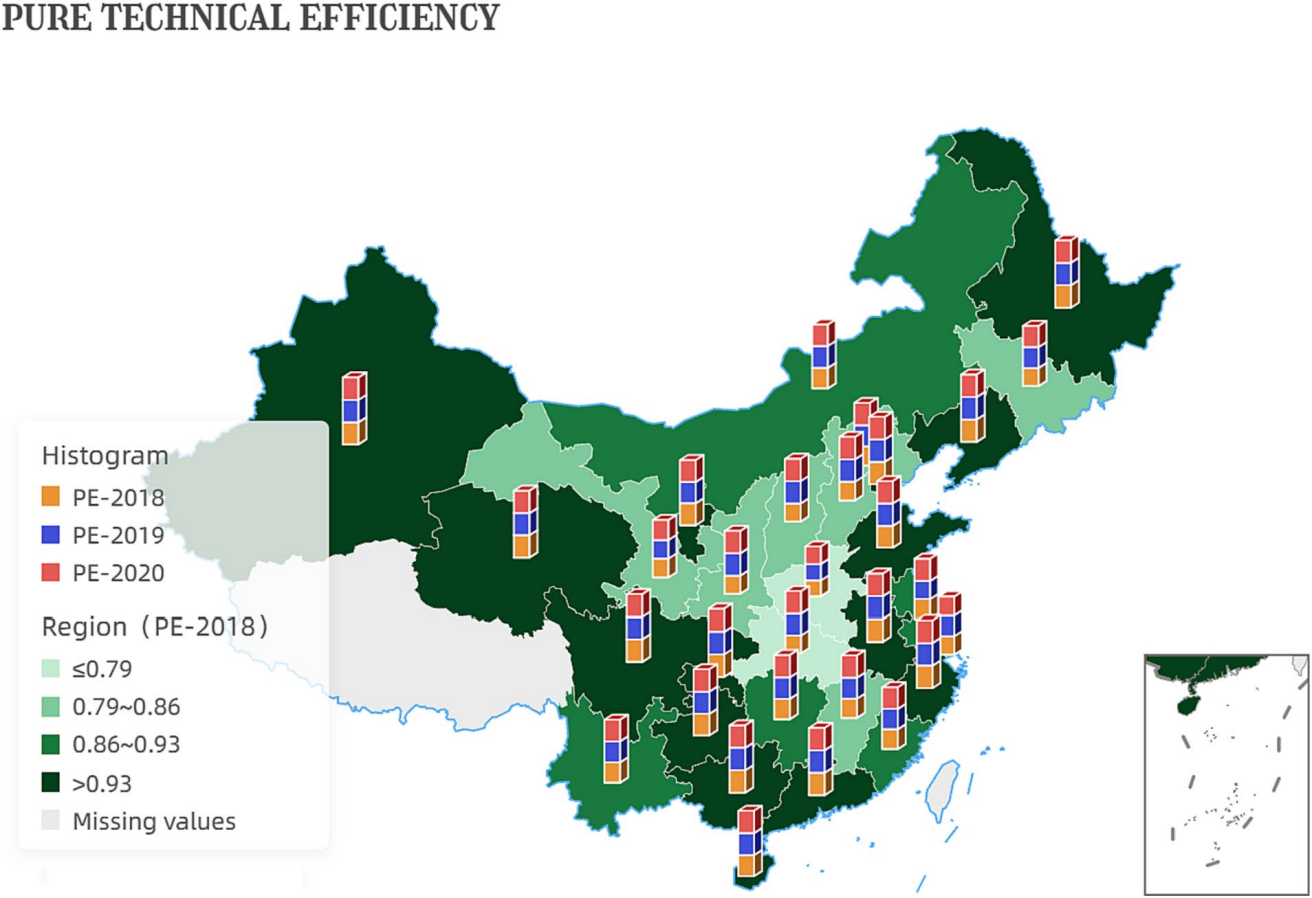
Development of inter-provincial pure technical efficiency.

Additionally, the increase in pure technical efficiency values indicates that the government has improved the quality and effectiveness of its services without increasing inputs. This improvement could be attributed to better training and skills development for government employees, improved information systems and communication channels, or more effective use of data and analytics to inform decision-making. Figure 5 shows the development of inter-provincial pure technical efficiency. China’s reform efforts to delegate power, streamline administration, and optimize government services have resulted in improved productivity and effectiveness of the government.

#### 4.3.2 Inter-provincial innovation efficiency cumulative distribution analysis

Drawing on the experience of Arvidsson (23) and Xue (24), who discuss DEA’s Innovation Efficiency Cumulative in the areas of urban resource allocation and tourism efficiency, it is evident that, since the deepening reform in delegating power, streamlining administration, and optimizing government services, provincial governments have strengthened macro management and services for the pharmaceutical manufacturing industry, simplified the related administrative procedures for approval, and subsidized R&D innovation, fully mobilizing the entire pharmaceutical industry’s enthusiasm for innovation. Pharmaceutical companies have more substantial confidence to invest their human and financial resources in innovation. The government and industry jointly optimize resource rationing and improve the efficiency of scientific and technological innovation in the pharmaceutical manufacturing industry.

**Figure 6:**
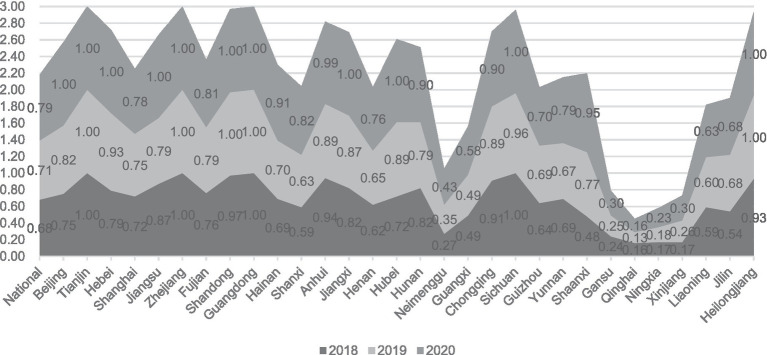
Efficiency accumulation chart.

Looking at the cumulative distribution of comprehensive technological innovation efficiency values of provinces across the country from 2018 to 2020, as shown in Figure 6, the cumulative comprehensive technical efficiency of Tianjin, Zhejiang, and Guangdong East provinces is 3, reaching the technological frontier surface every year. Based on the information available on the official government website, it can be concluded that three provinces are among the top 45 advanced manufacturing clusters. These provinces have been selected by the Ministry of Industry and Information Technology for their strong innovation support, presence of multiple newly registered pharmaceutical companies, and effective resource allocation.

Beijing, Hebei, Jiangsu, Shandong, Sichuan, Anhui, Jiangxi, Hunan, Hubei, Chongqing, and Heilongjiang, the 11 provinces to the deepening reform in delegating power, streamlining administration, and optimizing government services has been influential, the pharmaceutical industry innovation capacity improved year by year. However, there may be a surplus of human resources, a low conversion rate of industry-university research, poor marginal benefits of innovation in the pharmaceutical manufacturing industry, not timely formation of reasonable resource allocation, and other issues; the three-year cumulative comprehensive technical efficiency is slightly lower than Zhejiang and other provinces, in the 2.5 to 3 range, the second tier.

It is worth mentioning that the cumulative value of Shanghai’s comprehensive efficiency performed poorly, based on the characteristics of Shanghai’s large industrial scale, strong government support and high efficiency of services, and a good ratio of high-end technology, more likely lies in the inclusion of talent and capital and other resources overflow and lead to serious waste.

Fujian, Shanxi, Yunnan, Henan, Hainan, Guizhou, and Shaanxi, the seven provinces, do not have leading industries in the pharmaceutical industry, pharmaceutical distribution, and medical services. The number of high-quality pharmaceutical enterprises is small, and the output value is low. Although under the deepened reform in delegating power, streamlining administration, and optimizing government services, the pharmaceutical market has stimulated a certain amount of innovative vitality and improved innovation efficiency. Still, due to the scale, logistics, equipment, and other factors, the lack of competitiveness, the three-year cumulative comprehensive technical efficiency is low, reaching the 2–2.5 range, the third echelon.

Neimenggu, Guangxi, Gansu, Qinghai, Ningxia, Xinjiang, Liaoning, and Jilin are the eight provinces with three-year cumulative comprehensive technical efficiency below 2. The comprehensive technical efficiency of Gansu, Qinghai, Ningxia, and Xinjiang provinces is still below one cumulatively for three years. This reflects the sparse number of local pharmaceutical enterprises and the backwardness of the infrastructure related to the pharmaceutical industry. It is only possible to improve the efficiency of science and technology innovation in the pharmaceutical manufacturing industry in this province by relying on the deepening reform in delegating power, streamlining administration, and optimizing government services. It is more necessary to undertake high-quality pharmaceutical enterprises in other provinces. Increase cooperation and exchange with a more complete logistics system. To allow the further development of the pharmaceutical manufacturing industry.

## 5 Results and recommendations

This research uses a three-stage BCC model to measure and analyze the efficiency of science and technology innovation in the pharmaceutical manufacturing industry under the deep reform in delegating power, streamlining administration, and optimizing government services. Given the extensive influence of this reform, its effectiveness can be directly observed in enhancing the business environment, boosting the confidence of private enterprises, reshaping government functions, and elevating regulation and service standards. Therefore, it is essential to select input–output indicators that better reflect the effectiveness of the deepening reform in delegating power, streamlining administration, and optimizing government services of local governments after eliminating the environmental factors and random interferences that are indirectly related to input and output, such as talent education, economic level, people’s welfare, and industrial development. We obtained the comprehensive technical efficiency, pure technical efficiency, and scale efficiency of China’s inter-provincial pharmaceutical manufacturing industry under the deepening reform, which is consistent with reality. The following conclusions and recommendations are drawn:

The interprovincial integrated technical efficiency in the first stage is generally high, showing a trend of increasing efficiency year by year, and the interprovincial scale efficiency and interprovincial pure technical efficiency obtained by decomposition also perform well. However, environmental factors and random disturbances affect all three efficiencies. After the regression of the SFA model in the second stage, we found that the environmental factors affect the input–output as follows: increasing people’s welfare will increase the input efficiency of government subsidies and enterprise R&D personnel. It will lead to poor efficiency of enterprise development expenditure and input of Medical Products Administration; Industrial development helps the pharmaceutical manufacturing industry to save government subsidies and R&D personnel input to avoid waste; Improvement of economic level will make input from enterprise development expenditure and Medical Products Administration less wasteful, but there is redundancy of government subsidies and R&D personnel input; Improvement of talent education brings creativity to pharmaceutical manufacturing industry, but prone to poor allocation effect and inefficient development problems. In the third stage, after excluding the influence of environmental factors and random interference, inter-provincial comprehensive technical efficiency, inter-provincial pure technical efficiency, and inter-provincial scale efficiency show two characteristics:

- ① The provinces maintain the overall trend of increasing year by year;
- ② The value of each efficiency is significantly lower and decreases at different rates, which is more relevant to reality.

The overall trend of comprehensive technical efficiency growth shows the positive impact of the deepening reform in delegating power, streamlining administration, and optimizing government services in China’s inter-provincial pharmaceutical manufacturing industry. Measures have been effectively implemented to reduce micro-management and direct intervention and strengthen macro-control, market supervision, and public services. For individual provinces such as Jiangsu and Chongqing, where comprehensive technical efficiency first declines and then grows, attention should be paid to policy convergence and coherence to reduce the risk of policy fluctuations and instability in pharmaceutical enterprise innovation and to play a positive role in the group efforts of both government and enterprises.

The second feature shows that for most provinces in the western, central, and northeastern regions, the small scale of the industry and the single industrial chain are the key factors that hinder the efficiency of scientific and technological innovation in the pharmaceutical manufacturing industry. These provinces should attract foreign provinces or foreign investors to build factories, improve the scale of enterprises, and build supporting facilities. The state should play a macro-control role to change the current situation of uneven regional development of the pharmaceutical manufacturing industry give policy tilt and technical support to increase the innovation capacity of provinces with low comprehensive technical efficiency. In addition, if the allocation of resources can improve pure technical efficiency, Beijing, Shanghai, Jiangsu, Shandong, and other provinces can further play the advantages of solid pharmaceutical manufacturing, large economic output, and more talent.

## 6 Discussion and limitations

Since the creation of Data Envelopment Analysis (DEA) by Charnes, Cooper and Rhodes (7) in 1978, DEA has become an important theory and tool for analyzing efficiency in many fields. Examples include green finance and renewable energy (25), supplier selection in the steel industry (26), and heterogeneous R&D investment and green productivity (27). Another topic of interest to us is Mohsin’s (28). Data Envelopment Analysis (DEA), which analyses a panel of countries along the Belt and Road from 2008 to 2018 and assesses the relationship between public R&D expenditure and green economic growth and energy efficiency. Some other studies have assessed the impact of China’s Low Carbon City Pilot Program by introducing the quasi-experimental approach of the Malmquist-Luenberger productivity index in DEA and difference-in-difference propensity score matching (PSM-DID) (29).

We find richer DEAs regarding areas of science, technology and innovation. Zhang (30) measured the green innovation efficiency in the research and development (R&D) phase and the transformation phase of industrial technology in China (30 provinces and municipalities). Based on 278 prefecture-level cities in China, Liu (31) clarified the relationship and mechanism between technological innovation and green economic efficiency, and the results will promote the growth of green economy in emerging cities. Zhang (32) took China’s construction industry from 2000 to 2017 as an example to understand the impact of environmental regulation on the efficiency of green technological innovation in the construction industry.

In order to gain a deeper understanding of the technological innovation efficiency of China’s pharmaceutical manufacturing industry, we need to compare the previous results with those of Guo (33) and Tang (34). This will not only help us verify the accuracy of the study, but also reveal more valuable information.

The reform of delegating power, streamlining administration and optimizing government services has a significant impact on the efficiency of technological innovation in China’s pharmaceutical manufacturing industry. This is because the reforms have had a direct impact on the business environment and innovation capacity, providing strong support for the continued growth of the industry. However, it is worth noting that there are significant differences in the national contexts of different countries, which means that the strength of government authority and coercion varies from country to country. In addition, there may be significant differences between different provinces within the same country.

For reforms to be effective, these differences must be fully taken into account. This also means that the articulation and coordination of national and provincial policies is crucial. Only if policies remain stable, reduce unnecessary fluctuations, and break down information barriers can the reform truly play its intended role of promoting the continued efficiency of technological innovation in China’s pharmaceutical manufacturing industry.

## Data availability statement

The original contributions presented in the study are included in the article/Supplementary material, further inquiries can be directed to the corresponding author.

## Ethics statement

The studies involving human participants were reviewed and approved by the Ethics Committee of China Pharmaceutical University. Written informed consent for participation was not required for this study following the national legislation and the institutional requirements.

## Author contributions

YG: Writing – original draft. QZ: Writing – review & editing.
