# Supplementary material for: Research on the impact of China’s reform to delegate power, streamline administration, and optimize government services on the technology innovation efficiency of the pharmaceutical manufacturing industry
Source: Front Public Health. 2024 Jan 26;12:1325298. doi: 10.3389/fpubh.2024.1325298 (PMC10853378; doi:10.3389/fpubh.2024.1325298)
Supplement: Supplementary file 5 [file Table_5.DOCX]

**SFA regression analysis of the Second stage**

| environmental variables | Input slack-Subsidy | Input slack-  Material | Input slack-Account | Input slack-Intelligence |
| --- | --- | --- | --- | --- |
| Constant term | 2089.32** | -580989.09*** | -346948.46*** | 7978.66*** |
| People's welfare | -1262.90*** | 165750.37*** | 98333.51*** | -3024.27*** |
| Industrial development | -65.21 | -13474.32*** | -13215.00*** | -83.01 |
| Economic level | 805.52** | -30432.12*** | -15501.76*** | 1199.57*** |
| Talent education | -5.93 | 6474.55*** | 5609.91*** | 86.09 |
| **σ^2** | 131515.43 | 491039440.00 | 214534320.00 | 443779.65 |
| **γ** | 0.43 | 0.54 | 0.73 | 0.47 |
| **LR test of the one-sided error** | 9.44** | 12.18** | 42.15*** | 23.27*** |

Note: 1) ** and *** represent significant at the level of 5% and 1% respectively
